# Cohort Analysis of a 24-Week Randomized Controlled Trial to Assess the Efficacy of a Novel, Partial Meal Replacement Program Targeting Weight Loss and Risk Factor Reduction in Overweight/Obese Adults

**DOI:** 10.3390/nu8050265

**Published:** 2016-05-04

**Authors:** Emily Brindal, Gilly A. Hendrie, Pennie Taylor, Jill Freyne, Manny Noakes

**Affiliations:** 1 Food and Nutrition, Commonwealth Scientific and Industrial Research Organization (CSIRO), PO Box 10041, Adelaide, South Australia 5000, Australia; Gilly.Hendrie@csiro.au (G.A.H.); Pennie.Taylor@csiro.au (P.T.); Manny.Noakes@csiro.au (M.N.); 2 Health and Biosecurity, CSIRO, GPO Box 76, Epping, New South Wales 1710, Australia; Jill.Freyne@csiro.au

**Keywords:** meal replacement, diet, smartphone, weight loss, pharmacy

## Abstract

Our aim was to design and evaluate a weight-loss program, including a partial meal replacement program, point-of-care testing and face-to-face and smartphone app support, appropriate for delivery in a community pharmacy setting. Overweight or obese adults (*n* = 146, 71.2% female, 48.18 ± 11.75 years old) were recruited to participate in a 24-week weight loss study and randomised to two app conditions. The dietary intervention was consistent regardless of app. Twelve weeks of clinic appointments with a trained consultant were followed by only app support for an additional 12 weeks. By week 24, retention was 57.5%. There were no differences between app conditions. Based on a cohort analysis of the trial, the mean decrease in weight from baseline to week 24 was 6.43 ± 1.06 kg for males (*p* < 0.001) and 5.66 ± 0.70 kg for females (*p* < 0.001). Mixed models also revealed decreases for LDL Cholesterol (−0.13 ± 0.08 mmol/L, nonsignificant), triglycerides (−0.08 ± 0.05 mmol/L, nonsignificant) and an increase in HDL cholesterol (+0.08 ± 0.04 mmol/L, ns) were not significant by week 24. Blood glucose (−0.23 ± 0.08 mmol/L, *p* = 0.040) and blood pressure (Systolic blood pressure −5.77 ± 1.21 Hg/mm, *p* < 0.001) were significantly lower at week 24 compared to baseline. Weight loss self-efficacy increased and remained significantly higher than baseline at week 24 (16.85 ± 2.93, *p* < 0.001). Overall, the program supported participants and was successful in achieving significant weight loss and improvements in health outcomes over 24 weeks.

## 1. Introduction

There is evidence that meal replacements (MRs) are effective in facilitating weight loss in the context of intense multidisciplinary support. Hemysfield [1] conducted a meta-analysis comparing partial meal replacement programs (PMRPs) to traditional energy restricted food based diets, and found MRs resulted in a 7% loss in body weight at three months compared to 3% in traditional diets, which equated to about 2.5 kg greater weight loss at three months in the MR group. Overall, weight loss at one year was 7%–8% in the MR group compared to 3%–7% on traditional diets. At one year, 74% of the MR group had lost ≥5% of their body weight compared to 33% on traditional diets (*p* < 0.001). Partial meal replacement programs have also been related to better nutritional quality [2].

Weight loss success following a prescription of MRs is generally achieved with through multidisciplinary professional support and provided at no cost to participants in research studies. However, less is known about the effectiveness of MRs as a strategy for weight loss in real world settings. Meal replacements are now widely available to consumers without professional support or medical supervision, which is likely to affect treatment outcomes.

There is evidence that eHealth tools, such as mobile phone applications (apps) may be able to provide users with additional support while embarking on a MR, weight management program. Overweight and obese women on an eight week PMRP, engaged more with a supportive mobile phone app than a static information-based app, and as a result reported significant improvements in their mood over the study period. Weight loss in this group was approximately 3% of starting weight over eight weeks. While the differences in weight loss between the two app groups started to diverge over the length of the study, they failed to reach statistical significance by end of the eight week period [3]. Although apps provide some feelings of support, the provision of additional face-to-face support may be able to improve weight loss results.

The challenge remains to develop a model of care for a PMRP that can be delivered in a real world setting replicating the “consumer market”, but that still provides adequate structure and support for individuals to achieve longer term compliance and weight loss. Delivering programs within pharmacies has been one avenue pursued in an attempt to improve the reach of weight loss programs. Pharmacies can provide opportunistic advice, making the most of people’s presence in a community health care setting [4]. A review of pharmacy-based weight loss intervention programs reported weight losses of between 0.6 and 5.3 kg at three months, and longer term losses of 1–4 kg at 12 months [5]. There is currently another review underway to describe community-based pharmacy interventions for weight management, as well as smoking cessation and prevention of excessive alcohol consumption [6]. Two studies have explored the role of the pharmacy in the delivery of MR programs observing weight losses between approximately 3.5 and 4.5 kg at 6 months [7]. One describes the translation of an existing program to the pharmacy setting [8], the second a comparison of different weight loss approaches delivered in the pharmacy (MR *versus* reduced calorie diet) [7]. Both approaches resulted in weight loss and therefore support the delivery of weight management programs in a pharmacy. One study compared a range of weight management programs including commercial weight loss programs (such as Weight Watchers) and pharmacy-led one-to-one counselling [9]. Individuals participating in the pharmacy-based program who completed the trial lost 2.8 kg compared to 4–5 kg in various commercial programs. Weight loss in the pharmacy setting was not significant after one year and the authors observed that pharmacy staff were not as familiar with the concepts of weight loss as staff delivering the commercial programs. Weight loss outcomes within a pharmacy setting may improve if pharmacies delivered a commercial program with specialised trained staff, used MR products to create the energy deficit, and included the adjunct support provided by smartphone app.

This paper describes the development, delivery and results of a model of care for a weight management program using MRs, trained face-to-face support staff, point of care testing for risk factors and two versions of a mobile phone application to promote weight loss in a free living overweight/obese population. It was our objective to evaluate this program on its efficacy for weight loss, biological measures (blood pressure, capillary blood glucose, lipids) and self-efficacy based on observational changes over a 24-week period. Our secondary objective was to assess participation and evaluation data to indicate the program’s acceptability and feasibility.

## 2. Method

### 2.1. Study Design

Data were collected as part of a 24-week randomized controlled trial where participants were assigned to one of two study groups. Both groups followed a PMRP, with personalised advice on incorporating high-protein meal replacement shakes (manufactured by Probiotec Pty Ltd., Laverton North, Australia) and one high protein meal into their lifestyle. Both groups received support from a trained consultant, had access to a dietitian telephone hotline, and one of two iPhone apps. The apps were developed by CSIRO specifically to support this program. Different versions of the app differed in the number of monitoring tools and supportive features they contained to ascertain the value of different app features (data not reported). Both apps included detailed recipes for the program meals and detailed information about how to follow the program. Over 50 recipes and snack ideas were included under various headings (*i.e.*, seafood, beef, breakfast ideas *etc.*) to help users prepare balanced meals. One app was more interactive and also included self-monitoring tools, motivational and reminders in addition to the program information. Specifically it contained a weight tracker, a basic food diary and sent prompts to users asking them to enter their meal or weight data. It also presented users with a motivational message or thought for the day (e.g., don’t focus on your failures, learn from them).

The study included an active intervention period (12 weeks) followed by a 12-week free living period. During the intervention period, MRs were provided for the first 4 weeks, volunteers were required to purchase them at a nominal cost (1AUD per sachet) for the remainder of the study. This was done to improve the external validity of the results, as our objective was to evaluate not only observational changes in measures, but also the feasibility and acceptability of the program as a whole, which would ultimately involve provision of a product that would require the user to purchase it.

**Figure 1:**
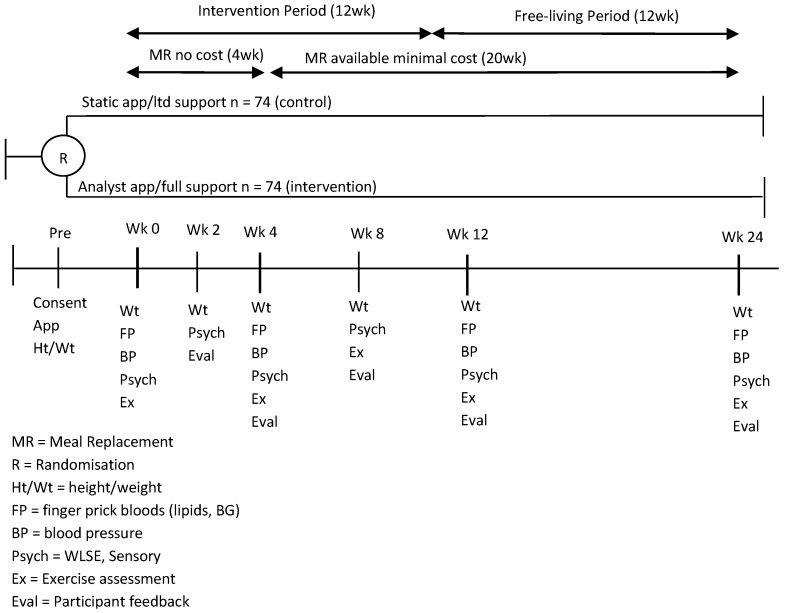
Study design over 24-week.

The study outcomes were collected at various time points (Figure 1). The study was approved by the CSIRO Food and Nutrition Human Research Ethics Committee and registered with the Australian New Zealand Clinical Trials Registry (registration number: ACTRN12613000547741).

### 2.2. Participants

Overweight or obese adults were recruited via an established clinic volunteer database and local media (news stories and paid advertisement in print media) between March and August 2013.

All potential participants completed a screening questionnaire which was reviewed by the trial manager to assess eligibility, including: Body Mass Index (BMI) greater than 25 kg/m^2^ (based on self-reported height and weight), aged 18 years and above, access to an iPhone, access to bathroom scales (to record their body weight), and willingness to participate in a PMRP and to have finger prick blood glucose/lipids assessed on four occasions. Exclusion criteria included pregnancy, uncontrolled diabetes, heart disease, or other medical conditions meaning participation in a MR weight loss study was not appropriate. All eligible participants provided written consent after attending an information session and were randomised to receive one of the iPhone apps by the trial manager using [10].

### 2.3. Study Measures and Outcomes

Primary outcomes were percent weight loss from baseline, and changes in biological measures including change in blood pressure, fasting blood glucose and fasting blood lipid (otal Cholesterol, LDL, HDL and Triglycerides) with secondary outcomes of weight-loss self-efficacy, changes in physical activity and participant feedback. Participant demographics were collected at enrolment to the study.

### 2.4. Program Delivery and Consultant Training

To better replicate a non-clinical, community pharmacy environment, study visits were conducted within booth-like consulting areas set up in our research institution with consultants who were allied health trained but not specifically trained in nutrition (1 had dietetics training, 1 psychology, 3 nursing). Where possible, individuals saw the same consultant throughout the trial. All consultants received program-specific training about the weight loss intervention, the decision support tool and how to perform point-of-care (PoC) testing. Training about the weight loss intervention and decision support tool was conducted by dietitians before the commencement of the study and at regular intervals throughout the first 12 weeks. Training on the process and procedure for conducting PoC measures was conducted by a nurse experienced in these methods.

#### 2.4.1. Point of Care (PoC) Measures

The program included PoC measures at baseline, and weeks 2 (weight only), 4, 12 and 24. These captured weight (kg) and the biological measures: blood pressure (Hg/mm), blood glucose levels (mmol/L), and blood lipid levels (mmol/L). Point-of-care measures are relatively quick to conduct and can provide immediate results for participants, allowing feedback to be provided at the time of taking the measures. Fasting blood glucose using an AccuCheck (Roche Diagnostics Australia, New South Wales, Australia) and lipid levels (Total Cholesterol, LDL and Triglycerides) using AccuCheck Trend Plus (Roche Diagnostics Australia, New South Wales, Australia) were measured once via a finger prick. Further measurements were taken if the reading fell outside the acceptable range or a machine error was received. Two blood pressure measurements were taken using an Omron (Omron, Osaka, Japan). Participants were asked to be seated for 5 min prior to the first measurement; a subsequent reading was taken two minutes later. A third measure was only taken if the difference between readings fell outside an acceptable range (*i.e.*, Systolic within 10 mmHg).

#### 2.4.2. Weight-Loss Self-Efficacy, Physical Activity and Participant Feedback

The 20-item weight-loss self-efficacy scale (WLSE) was used to assess individuals’ confidence in their ability to control their eating in a variety of situations [11]. Questions were asked on a 10-point scale and summed to create a total WLSE score (0–180). Higher scores indicate greater feelings of efficacy to control eating. At baseline, the scale showed strong internal consistency (Cronbach’s α 0.95).

Physical activity levels were measured using the short form of the international physical activity questionnaire (IPAQ-SF; available at [12]). In order to capture general levels of activity, Metabolic Equivalent of Task (MET) minutes per week were calculated at each time point for each individuals as per the instrument scoring instructions. Sitting time (minutes per week) was calculated independently of MET minutes.

From week 2 onwards, participants were asked to rate their liking of the MR shakes and their current desire to consume them. These were both answered on a 10-point scale with the end points being 1 “one at all” and 10 “very strong”.

Finally, participants were asked a range of open-ended evaluation questions to capture their experience on the program. The questions relevant to this paper included, “What has been the most helpful aspect of the program” asked at week 12 and “Please add any other comments you wish to make specifically about the meal replacement shakes” which was asked at week 2.

## 3. Weight Loss Intervention

### 3.1. Program Description

The weight management program was designed as a prototype version of the Impromy program. It was a PMRP designed to transition people toward a balanced, whole food diet. To do this, the program included three phases: Motivate, Advance, and Sustain. The Motivate and Advance phases contained three levels, which varied in the number of MRs and food-based snacks prescribed based on participant BMI. The Motivate phase was designed to result in an energy deficit of approximately 30% of energy requirements. The three levels provided 5000 kJ (BMI = 25–30), 6000 kJ (BMI = 30–35) and 7000 kJ (BMI ≥ 35), and consisted of 2–4 MRs, 1 higher protein balanced meal and 2 snack units. The Advance phase was designed for an energy deficit of about 20%, with fewer meal replacements and more food based meals and snacks prescribed. The Sustain phase was designed for weight maintenance with no energy deficit applied. This phase comprised of 3 main meals plus 3 snack units, with the option to use a MR as a substitute for a main meal when convenient or at the individuals’ discretion.

Progression from Motivate through to Advance or Sustain was a joint decision between the individual and consultant depending on their weight loss success, goal weight, readiness to change, fatigue with meal replacements, current motivation and life events. These questions were presented using a set of standardised “if/then” questions and answers described in a matrix, referred to as, the “decision support tool”. The decision support tool was developed by the study team to improve the consistency of program delivery between consultants and increase intervention integrity. The questions within this decision support tool were based on experience with previous weight loss trials, and included questions relating to potential barriers to follow the program such as dislike of meal replacements, boredom with the diet plan, work/life commitments; reasons for accelerated or prolonged time in a phase such as slower than expected weight loss, greater weight loss goal; or suggested modifications to the program to address issues such as hunger, social functions, or increase in activity. Points of discussion and potential solutions were included for the consultant to discuss with the individuals and a joint decision for progression agreed prior to completion of the visit. All consultants received training from the research dietitians in using this tool and were updated on the refinements throughout the study.

The 42 g MR shake powders were recommended to be made with 250 mL of low fat milk resulting in a composition as consumed of 1076–1122 kJ (vanilla or chocolate), providing 24.9–25.6 g of protein, 4.1–5.1 g of fat, 26.5–26.7 g of carbohydrates, plus 5.9–6 g of fibre. They were designed in collaboration with a manufacturer (Probiotec Pty Ltd.) in order to ensure they met Food Standards for Australian and New Zealand guidelines (Standard 2.9.3; [13]).

A variety of high protein meal recipes were provided in the iPhone app each providing about 1700 kJ, and 45 g protein with vegetables. Snacks were all food-based providing about 500 kJ (e.g., 2 small pieces of fruit, a small tub of yoghurt, a small muesli bar, a slice of raisin toast). A free foods list and list of indulgences was also provided. Free foods included whole foods with a low energy density such as low starch vegetables, clear soups, unflavoured mineral waters, and low kJ condiments. Indulgence foods provided about 600 kJ, were high in sugar, salt or fat and included confectionary and chocolate bars, ice cream, salty snacks and alcohol.

### 3.2. Program Support

At each visit, individuals were weighed. Their weight loss progress and working through the decision support tool formed the basis of their discussion with the consultant. Program information, nutrition education and recipes were provided through the app, regardless of the initial study group.

### 3.3. Analysis

Mixed models were used to assess data over time while controlling for any effects of app condition, participant sex, age and length of dieting history (effects of control variables not reported). The interaction between sex and week (time point) was also included in the model. Mixed model analyses use all available data at each time point and are considered an intention-to-treat approach to analysis. Outcomes analysed in these models included weight, systolic and diastolic blood pressure, total cholesterol, triglycerides, LDL, HDL, blood glucose, liking and desire to consume the MRs, MET minutes of exercise per week, sit minutes per week and weight-loss self-efficacy. Significant main or interaction effects were followed with pairwise, *post hoc* comparisons using Bonferroni adjustments. Statistical Package for the Social Sciences (SPSS) version 20 (IBM, Armonk, NY, USA) was used for all analysis. Significant alpha levels were set at *p* < 0.05.

Program feedback was based largely on open-ended response questions. These were generally brief and a research psychologist coded these responses according to key themes. This process involved coding the data at several levels of details until themes were consistent and allocations to groups were reliable.

## 4. Results

### 4.1. Participants

A majority of participants were female, obese category 1 or 2, employed and had owned an iPhone for over 12 months (Table 1). Previous diet experience was varied, as was their experience with meal replacement products. Of the participants who had never used meal replacements, most (61.5%) had dieted 5 times or less. Most of the sample had tried some form of dieting in the past but 65.1% had never or rarely used meal replacements.

**Figure 2:**
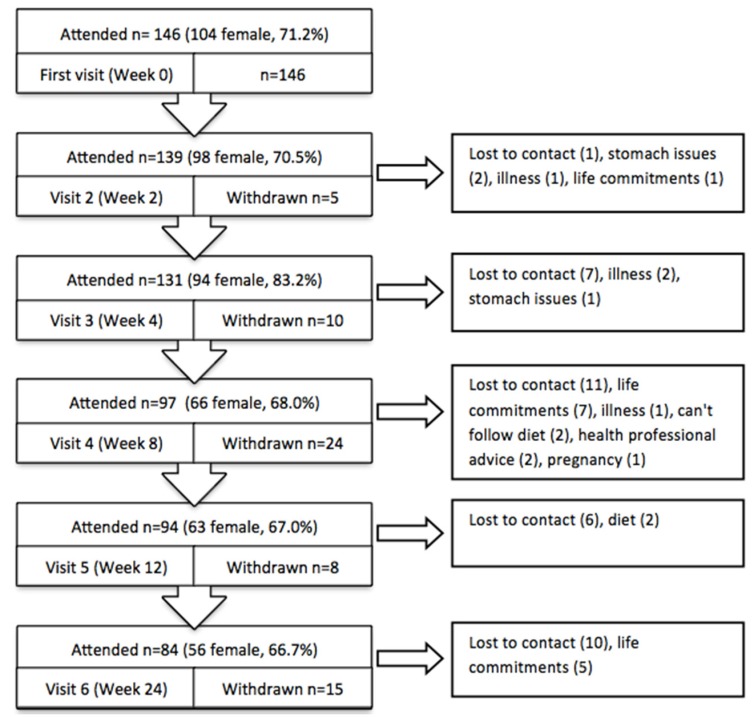
Study sample flow and withdrawals. Note: some people failed to attend on visit but did not withdraw from the study.

At the end of the study, there was no difference in drop-out between the two app groups (χ^2^(1, 145) = 0.96, *p* = 0.547). By Week 24, 57.5% of participants remained in the study (Figure 2). The large drop-out (*n* = 24) between weeks 4 and 8 corresponded with the cessation of the provision of free meal replacements. A majority of drop-outs were lost to contact with no reason provided (*n* = 35, 56.5%). A small portion (*n* = 7; 11.3%) identified gastrointestinal issues or difficultly with the diet as the reason for withdrawal.

### 4.2. Weight Loss Results

**Figure 3:**
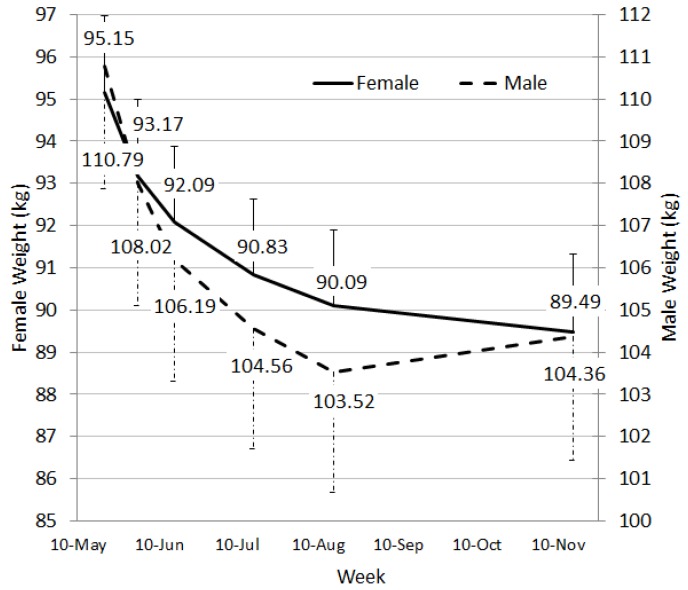
Adjusted weight loss values (kilograms) through the trial period. Error bars represent 1 standard error.

The interaction between sex and week for weight was significant with males being significantly heavier than females at each time point (F(5, 110.0) = 6.67, *p* < 0.001). The mean difference in weight between week 24 and baseline was −6.43 kg (SE 1.06, *p* < 0.001) for males and −5.66 kg (SE 0.70, *p* < 0.001) for females. Males and females had subtly different patterns in weight loss (Figure 3). The core difference being that females’ weight fell more or less consistently, whereas males’ weight fell until week 12, but began a small, non-significant rebound at week 24. For both groups there were not significant changes between week 12 and 24.

Amongst only completers (*n* = 84), percentage weight loss from baseline to week 24 ranged from +3.3% to −22.79% with 58.4% losing 5% or more of their initial weight. This represents 33.5% of all starters (*n* = 146). Of those losing 5% or more, 51% lost 10% or more of their starting weight. By week 24, 51.8% of participants had transitioned to the Advance phase, with 28.9% on Sustain and the remainder stayed in the Motivate phase.

### 4.3. Biological Measures

Systolic and diastolic blood pressures were significantly different by study week (Table 2). Between baseline and week 24, the difference was a decrease of 2.69 ± 1.04 (*p* > 0.05) for DBP and 5.77 ± 1.21 for SBP (*p* < 0.001). Systolic blood pressure values fell consistently with comparisons between baseline and other weeks significant (all *p* < 0.001). Diastolic blood pressure was only significantly lower than baseline at weeks 4 and 12 (*p* ≤ 0.030).

Aside from values at baseline and week 24 (mean difference 0.12 ± 0.12, *p* > 0.05), total cholesterol values differed significantly at each week (all *p* ≤ 0.022). This represented a significant decrease in cholesterol followed by a rebound. However, values at week 24 did not significantly exceed the initial ones. Other blood lipid markers followed a similar pattern to total cholesterol aside from HDL cholesterol which had a higher non-significant value at week 24 compared to baseline (mean increase 0.08 ± 0.04, *p* > 0.05).

The only significant pairwise comparison for blood glucose by week was between baseline and week 24 which represented a significant decrease (mean difference 0.23 ± 0.09, *p* = 0.040) (Table 2).

### 4.4. Activity Levels and Subjective Assessments

There were no significant changes for MET minutes of activity or sitting time per week (Table 2).

The trial week significantly affected weight loss self-efficacy (Table 2). Weight loss self-efficacy rose and then gradually declined throughout the trial but remained significantly higher than baseline at each time point (*p* < 0.001).

**Figure 4:**
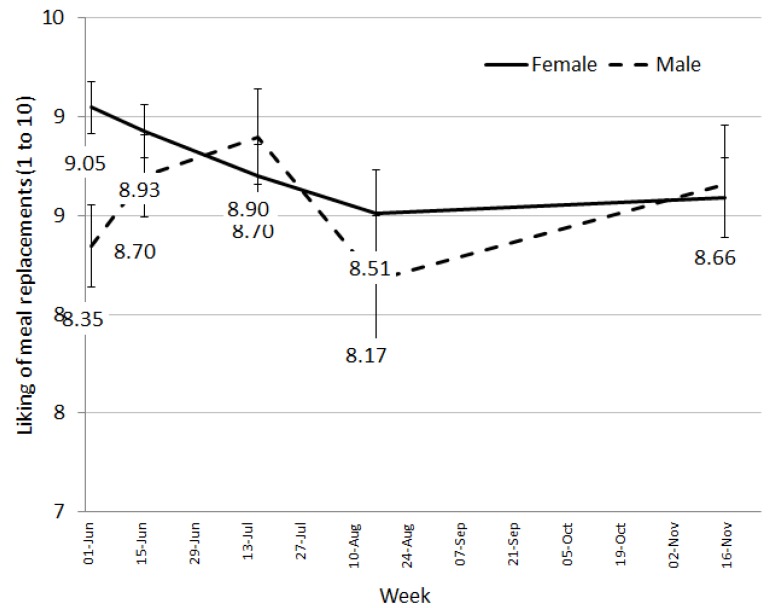
Adjusted means for liking presented by sex and week.

**Figure 5:**
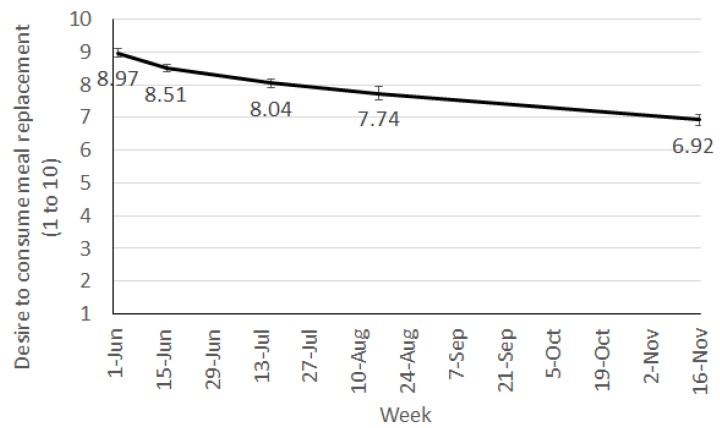
Adjusted means for desire to consume meal replacements.

Week significantly affected desire to consume (F(4, 95.3) = 10.33, *p* < 0.001) and liking of MRs (F(4, 91.0) = 2.94, *p* = 0.025). The desire to continue having MRs fell throughout the 24 weeks and was significantly lower at week 24 compared to all other weeks (Figure 4). Liking remained relatively high through the 24 weeks (Figure 5). The significant interaction between week and sex (F(4, 91.0) = 2.94, *p* = 0.025) revealed that males and females had different liking patterns over time (Figure 5). Liking scores from females fell consistently from week 2 to week 24 with ratings of liking significantly lower by week 24. Males had a less clear pattern in ratings of liking over time with an initial peak, followed by a fall at week 12.

When asked an open-ended question about the most helpful elements of the program 59.9% of comments were about the core program components—the meal replacements, the face-to-face support or the smartphone app. A small group of people offered no suggestions (5.1%) and a similar amount also described their own personal attributes (such as being motivated or disciplined). Only 3.7% specifically mentioned seeing the results of their blood indicators (Table 3).

General feedback on the meal replacements was provided by 72 participants who offered 94 individual comments on the meal replacements. There were 57 comments with a positive inclination (*i.e.*, were mentioned in the context of being “great” or “liked”). These referred to taste (*n* = 16), general liking (*n* = 12), consistency of MR (*n* = 8), ease of use (*n* = 7), feeling full (*n* = 3), using milk rather than water (*n* = 3) and general convenience (*n* = 2). Other comments (*n* = 6) were made by only single individuals. Sixteen comments related to negative aspects of the shakes, only consistency of MR (*n* = 4) and increases in gastro-intestinal wind (*n* = 5) were mentioned multiple times. Remaining comments were not given with enough detail to assess whether they were positive or negative.

## 5. Discussions

Overall, the model of care using trained consultants, a PMRP and a mobile phone app was successful with both males and females losing between 5 and 6 kg over a 24 week period. Of all starters, 33.5% lost a clinically significant amount of weight. Other studies have reported similar success in weight loss for PMRPs. For example, in a pharmacy-delivered weight loss program, Ahrens *et al.* prescribed two meal replacements daily and reported an average weight loss of 4.90 kg over 12 weeks [7]. A second example of a PMRP transitioned participants from two to one meal replacements after 3 months [14]. An average weight loss of 7.36 kg was reported at 12 weeks, and almost 9 kg at 12 months. While this study is similar to ours in their transition approach, their attrition was low (32.7% at 12 months), the program was delivered by clinical trial staff through an Obesity Clinic and included participants referred by a physician. Our weight loss results were slightly lower (but sustained to 6 months), and also achieved in a self-selected sample of participants who received limited personal contact of six visits and additionally had to purchase their MR.

Reductions in weight were also accompanied by significant decreases in blood pressure, and small, consistent decreases in blood glucose which became of significant magnitude by week 24. Total cholesterol, LDL and triglycerides fell significantly between baseline and week 4, but generally rebounded, and by week 24 changes in total cholesterol were not different to baseline. This may be because people starting transitioning to less daily MRs at week 4. Other authors have reported similar changes in blood lipids associated with active weight loss, followed by rebound during a follow up or maintenance phase [7]. There were no changes in physical activity level of participants, but we did see an improvement in weight loss self-efficacy. A person’s confidence in their ability to perform the behaviours necessary to maintain weight has been associated to the behaviours they perform [15] and therefore could be an important factor for weight loss success.

Weight loss interventions are known to have relatively high attrition rates, because complying with a new dietary regime and the time commitments associated with participation can be difficult. However, at week 12 almost 65% of participants were still involved in our program and after 24 weeks 58% of people who started the program were still actively involved. Similar retention has been reported in other meal replacement or pharmacy based weight loss trials. In a pilot trial of a PMRP with app support but without any face-to-face support, retention was 76% at week at 8 [3], whereas the equivalent for the current study was 83.5%. Our 64.4% retention at 12 weeks was similar to other studies in a pharmacy not including meal replacements (62.9%) [9], but better than a pharmacy-delivered community program (56.0%) [8]. Although on face value, our retention seems similar, these values were achieved when participants had to purchase the meal replacement product. There is some evidence that provision can drive retention as well as weight loss [16], but others suggest providing free food over and above a structure meal plan provides no further benefit [17]. Our large decrease in retention after week 4 supports suggestion that the provision of free products motivated some people to stay engaged with the program. Despite this we retained a respectable proportion of participants at 6 months. As part of the study design, we created a pharmacy-like environment and employed casual staff, but it is important to acknowledge that the study was conducted within a research institution and participants may have had higher commitment to the program due to its relationship with the scientific trial.

The MR product used was manufactured specifically for this trial and only two flavours were available—chocolate and vanilla. Although the literature would suggest that sensory specific satiety may have become an issue [18], liking of the meal replacement shakes remained high throughout the trial (8/10). This is extremely promising as liking is a critical factor for food choice [19] and therefore, continuing persistence on the diet. Early in the trial (at week 2 with 96.6% of participants remaining), we asked for feedback specifically on the meal replacements and received over 60% positive comments with only 17% negative comments. The negative comments related to increases in gastro-intestinal wind possibly due to changes in fibre or general dietary restrictions, the amount of milk being ingested, or the consistency of the shake, which may be due to how the shake was prepared by the individuals.

Despite generally high liking ratings, participants’ desire to consume the meal replacement shakes did decrease steadily throughout the trial, suggesting a fall in their motivation to continue having the shakes. This may be an early indicator of falling compliance and discontinuation of the diet. For longer term success on a program such as this, providing individuals with the flexibility to transition through to fewer meal replacements as their weight loss progresses or as fatigue with the shakes sets in becomes an important element for success. Pharmacy staff are ideally placed to assist the community with weight loss as they are readily accessible and can be available to consultant with individual’s on an as needs basis, potentially quicker that seeking advice from other health professionals. However, appropriate training and tools are required to ensure pharmacy staff delivering the program (not qualified in nutrition) have adequate support to facilitate such a transition through a weight loss program.

The feedback that we received on the program was largely positive with most people identifying core program components such as the meal replacements, consultant and phone app support, as the most helpful elements. The blood measures received few positive comments. Participants may not have seen these as elements of the program and thought they were part of the study outcomes. The simplicity of MRs means they are an easier intervention to use in this type of program. Participants reported to like this, in combination with enhanced support. Comments on the face-to-face contact and the app made mention of increased accountability—“being accountable to someone or something”. This was interesting as previous studies have indicated that both personal contact and technology could have roles at different stages of a weight loss program with technology being more important early and personal contact featuring later [20]. Together the elements that we have tested have the potential for roll-out into a free-living market with the pharmacy, app and consultant components all designed to be easily translated and up-scaled.

Results of our study were limited by the observational nature of the data analysed. There was no comparison group or ‘standard’ weight loss program to compare the effectiveness of our program to. The use of mixed model analyses allowed us to use all available data in an intention to treat approach, which is preferred in clinical trials with repeated measures on individuals over time. We also recruited a higher number of women than men, and our attrition rate was over 40%, although both of these are common limitations in weight loss and nutrition research. We collected limited data on total diet and do not know if general dietary patterns changed throughout the trial. It may be useful to explore this in future trials. Finally, PoC outcomes were part of the program as well as a study outcome. Collecting intravenous blood may have resulted in more reliable measurement of risk markers. However, this would have interfered with the delivery of the program and the machines we used are often used in clinical settings and considered some of the most accurate.

## 6. Conclusions

Our results provide a promising evaluation of this integrated model of care combining high protein MRs and meals with a phone app, point-of-care health testing and personalised support from a trained consultant. However, there are areas of the program we would refine in future iterations. For example, increasing the energy restriction may accelerate greater weight loss in the initial phases of the program, and early weight loss has been associated with greater long term success [21]. The current program provided limited detail on exercise and a more detailed exercise program may also facilitate better long term weight maintenance [22]. A future trial may also directly compare the effectiveness of our program to an existing or ‘standard’ weight loss program in terms of long term weight loss. Nonetheless, results reported here are very encouraging for this novel weight loss program, for which participants who stayed on the program were positive, engaged and, most importantly, experienced weight loss success. Future research will focus on increasing retention to in turn increase the number of people achieving weight loss success.

## Figures and Tables

**Table 1:** Participant demographics for those starting the trial (*n* = 146).

Demographic	Option	*n*	%
**Sex (Female)**		104	71.2
**BMI Category**	Overweight (25–30)	27	18.5
	Obese I (30–35)	51	34.9
	Obese II (>35)	68	46.6
**Work Status**	Unemployed	5	3.4
	Student	2	1.4
	Retired	15	10.3
	Part-time	36	24.7
	Full-time	77	52.7
	Casual	4	2.7
	Mixed	7	4.8
**Lifetime Diet History**	Never	8	5.5
	1–5×	49	33.6
	6–10×	38	26.0
	11–15×	10	3.8
	>15×	41	28.1
**Education Level**	Below Secondary School	2	1.4
	Secondary School	39	26.7
	Technical Qualifications/Diploma	55	37.7
	Bachelor’s Degree	28	19.2
	Post-graduate Degree	22	15.1
**IPhone Ownership Duration**	<1 month	5	3.4
	1 to 3 months	4	2.7
	3 to 6 months	10	6.8
	6 to 12 months	16	11.0
	>12 months	111	76.0
**Previous Use of Meal Replacements for Dieting**	Never	52	35.6
	Rarely	43	29.5
	Sometimes	50	34.2
	Always	1	0.7
**Age (*M* ± SD)**		48.18	11.75

**Table 2:** Adjusted means with standard errors (*M* ± SE) based on mixed models for study measures reported by study week and main model effects for week.

Measure	Standard Errors	Week 0	Week 4	Week 12	Week 24	Week Main Effect
SBP (Hg/mm)	*df*	140.5	136.5	105.4	98	F(5, 100.5) = 15.86, *p* < 0.001
	*M* ± SE	129.46 ± 1.29 ^a^	123.33 ± 1.16 ^b^	123.12 ± 1.43 ^b^	123.70 ± 1.29 ^b^	
DBP (Hg/mm)	*df*	141.6	131.5	104.4	91.4	F(3, 97.9) = 5.21, *p* = 0.002
	*M* ± SE	80.53 ± 0.83 ^a^	77.52 ± 0.97 ^b^	77.79 ± 0.97 ^b,c^	77.84 ± 1.14 ^a,b,c^	
Blood Glucose (mmol/L)	*df*	144.0	135.2	104	101.4	F(3, 95.0) = 3.22, *p* = 0.026
	*M* ± SE	4.66 ± 0.09 ^a^	4.56 ± 0.06 ^a,b^	4.55 ± 0.07 ^a,b^	4.42 ± 0.07 ^b^	
Cholesterol (mmol/L)	*df*	140.8	133.9	115.2	97.6	F(3, 102.5) = 34.92, *p* < 0.001
	*M* ± SE	4.64 ± 0.11 ^a^	3.91 ± 0.08 ^b^	4.27 ± 0.08 ^c^	4.53 ± 0.09 ^a^	
Triglycerides (mmol/L)	*df*	139.9	139.9	119.1	92.3	F(3, 101.6) = 5.09, *p* = 0.003
	*M* ± SE	1.19 ± 0.06 ^a^	1.03 ± 0.04 ^b^	1.12 ± 0.05 ^a,b^	1.10 ± 0.05 ^a,b^	
LDL Cholesterol (mmol/L)	*df*	137.1	134.5	111.51	101.46	F(3, 95.0) = 13.83, *p* < 0.001
	*M* ± SE	2.79 ± 0.08 ^a^	2.39 ± 0.07 ^b^	2.66 ± 0.08 ^a^	2.66 ± 0.08 ^a^	
HDL Cholesterol (mmol/L)	*df*	143.2	134	102.8	103.2	F(3, 102.05) = 32.48, *p* < 0.001
	*M* ± SE	1.28 ± 0.04 ^a^	1.08 ± 0.03 ^b^	1.16 ± 0.03 ^c^	1.36 ± 0.04 ^a^	
Sitting Minutes (/week) ^§^	*df*	143.7	128.3	54.7	78.0	F(4, 75.4) = 0.66, *p* = 0.621
	*M* ± SE	406.83 ± 32.82	345.19 ± 34.48	355.30 ± 36.62	415.47 ± 112.16	
MET Minutes (/week) ^‡^	*df*	142.2	130.5	103.5	88.8	F(4, 101.8) = 0.66, *p* = 0.621
	*M* ± SE	5059.67 ± 243.46	5059.88 ± 246.51	5417.05 ± 306.29	5394.52 ± 293.05	
WLSE (0–180) ^†^	*df*	154.3	138.3	126.9	125.9	F(4, 100.9) = 23.42, *p* < 0.001
	*M* ± SE	115.30 ± 2.98 ^a^	142.02 ± 2.61 ^b^	136.84 ± 3.13 ^b,c^	132.15 ± 3.52 ^c^	

Note: values without shared superscript letter are significantly different by week based on Bonferroni-adjusted pairwise comparisons (*p* < 0.05). ^a^ Week 8 values *df* 73.1; M ± SE 396 ± 52.58; ^b^ week 8 values *df* 107.5; M ± SE 5092.31 ± 305.38; ^c^ week 8 values *df* 124.8; M ± SE 141.33 ± 3.12; *p* < 0.05 for comparisons between week 8 and weeks 0 and 2; *df*, degrees of freedom; SPB, systolic blood pressure; DPB, diastolic blood pressure; MR, meal replacement; ^§^ no significant changes ^‡^ MET, Metabolic Equivalent of Task, no significant changes; ^†^ WLSE, weight-loss self-efficacy.

**Table 3:** Percentage of different themes of open-ended responses about most helpful aspect of the program received at week 12 of the trial.

Theme	Percentage
Accountability/Support	5.1%
App	15.3%
Shakes	25.6%
Face-to-face contact/Staff	19.0%
General awareness	4.4%
Bloods results	3.7%
Mindset (own)	5.8%
Recipes	2.9%
Nothing/no comment	5.1%
Structure/simple	8.8%
Information provided	4.4%

Note: Based on the 137 comments from 90 participants.

## Abbreviations

The following abbreviations are used in this manuscript:

App	Application
MR	Meal replacement
PMRP	Partial Meal Replacement Program
BMI	Body Mass Index
LDL	Low-density lipoprotein
HDL	High-density lipoprotein
PoC	Point-of-care
MET	Metabolic Equivalent of Task
WLSE	Weight-loss self-efficacy
